# Generalized Linear Mixed Effects Modeling (GLMM) of Functional Analysis Graphical Construction Elements on Visual Analysis

**DOI:** 10.1007/s40614-024-00406-4

**Published:** 2024-04-30

**Authors:** Art Dowdy, Kasey Prime, Corey Peltier

**Affiliations:** 1 Department of Teaching and Learning, College of Education and Human Development, Temple University, Philadelphia, PA USA; 2 Department of Educational Psychology, Jeannine Rainbolt College of Education, University of Oklahoma, Norman, OK USA

**Keywords:** Functional analysis, Multielement design, Visual analysis, Open science, Graph construction, DPPXYR, GLMM

## Abstract

Multielement designs are the quintessential design tactic to evaluate outcomes of a functional analysis in applied behavior analysis. Protecting the credibility of the data collection, graphing, and visual analysis processes from a functional analysis increases the likelihood that optimal intervention decisions are made for individuals. Time-series graphs and visual analysis are the most prevalent method used to interpret functional analysis data. The current project included two principal aims. First, we tested whether the graphical construction manipulation of the x-to-y axes ratio (i.e., data points per x- axis to y-axis ratio [DPPXYR]) influenced visual analyst’s detection of a function on 32 multielement design graphs displaying functional analyses. Second, we investigated the alignment between board certified behavior analysts (BCBAs; *N* = 59) visual analysis with the modified visual inspection criteria (Roane et al., *Journal of Applied Behavior Analysis*, *46*, 130-146, 2013). We found that the crossed GLMM that included random slopes, random intercepts, and did not include an interaction effect (AIC = 1406.1, BIC = 1478.2) performed optimally. Second, alignment between BCBAs decisions and the MVI appeared to be low across data sets. We also leveraged current best practices in Open Science for raw data and analysis transparency.

Functional analysis (FA) technology is an iterative assessment process to identify environmental variables that occasion and maintain open or closed contingency classes of behavior (Jessel et al., 2022) with the intent to develop function-based treatment (Beavers et al., 2013). Iwata et al. (1994) proposed the first comprehensive model of functional analysis with nine individuals with intellectual disabilities who engaged in self-injurious behavior. Four conditions were included: (1) a play or control condition; (2) social negative reinforcement in the form of presenting demands; (3) social positive reinforcement in the form of attention; and (4) sensory or automatic behavior (Iwata et al., 1994). This model of functional analyses has been replicated across hundreds of studies (Beavers et al., 2013; Hanley et al., 2003).

Advancements in FA formats have been developed with the intent to optimize efficiency, feasibility, and accuracy when identifying contingencies that occasion and maintain behavior (Rajaraman et al., 2022; Saini et al., 2020). Examples of advanced FA formats consist of (1) the brief FA (Northup et al., 1991); (2) latency-based FA (Thomason-Sassi et al., 2011); (3) trial-based FA (Bloom et al., 2011); and (4) the interview-informed synthesized contingency analysis (Hanley et al., 2014). Although copious research on FA technology has occurred since its inception, one characteristic that has remained constant is that FAs generally involve experimentation to test conditions on behavior, and in turn these data are presented on a time-series graph using single case experimental design (SCED) and are interpreted using visual analysis (Kinney et al., 2022). The multielement design is the most frequently used SCED for functional analyses (Desrochers & Fallon, 2014; Saini et al., 2020).

The evolution of FA technology has allowed for the precise identification of an individual’s function of behavior or behavior class (Warner et al., 2020). A unique aspect of FAs through the use of SCED is the ability for researchers to share raw data from the experiment via a time-series graph. This transparency allows for the analysis of data to make decisions about assessment outcomes and intervention selection. In SCED, visual analysis was the historical and still most frequently used approach to evaluate the effectiveness of interventions (Ledford et al., 2018; Tanious & Onghena, 2021). Despite visual analysis being the most frequently applied data analytic approach for SCED (Tanious & Onghena, 2021), there are mixed findings regarding the reliability of this method (Ninci et al., 2015; Wolfe & McCammon, 2022).

Ninci et al. (2015) conducted a meta-analysis on interrater agreement between visual analysts of SCEDs. The overall weighted interrater agreement was .76, however, there was heterogeneity in observer agreement across studies. There was a subsample of eight studies including 783 data sets displaying multielement designs and the mean proportion agreement was .80, which was greater than other design types. A closer look at primary studies included in the review highlights that visual aids with training can enhance agreement. Bailey (1984) identified the inclusion of a visual aid on the graph enhanced agreement amongst novice visual analysts (.85, .87) compared to graphs without a visual aid (.70, .74). Likewise, Roane et al. (2013) identified a visual aid and training led to high agreement among experienced and novice visual analysts evaluating multielement designs displaying FA results (range = .92–.98) compared to visual aid without training (range = .73–.80).

In a recent investigation not included in the prior review, Rader et al. (2021) evaluated the accuracy, reliability, and bias in judgments of functional analyses. A total of 121 doctoral level board certified behavior analysts (BCBA-D) who were experienced in visual analysis evaluated 10 FA graphs. Data were collected using signal detection theory on visual analysts’ interpretation of functional analysis graphs. Namely, extraneous variability represented noise in included graphs and the true function represented the signal. Visual analysts were evaluated on whether they detected a signal against the background of noise. Results showed that when 13 outliers were removed, 108 attentive participants produced a modest *d* of 1.59 analogous, which resulted in 63% accuracy. In signal detection theory (SDT), *d* is a measure of discriminability. A greater *d* indicates a better ability to discriminate between the signal and noise and a *d* of 0 indicates that the observer cannot distinguish between them. Rader et al. concluded that low accuracy across raters was likely due to insensitivity in detecting the signal from the noise. In addition, when the data were slightly elevated compared to the control condition, visual analysts were not able to detect the difference. Overall, these findings suggested that reliability of visual analysis was modest, even among experts.

To support visual analysis, structured approaches to visual analysis have been proposed for evaluating data collected using multielement designs. Hagopian et al. (1997) proposed placing criterion lines approximately 1 *SD* above and below the mean of the play condition and evaluating the percentage of data points outside this bandwidth per condition to determine function. Roane et al. (2013) extended upon Hagopian et al. and proposed the modified visual inspection (MVI) criterion—the main contribution was the requirement of 10 data points per condition was removed. Saini et al. (2018) proposed an extension to the MVI, ongoing visual inspection (OVI). OVI mirrors Roane et al. (2013) except it is used in a formative process during the experimentation process rather than a post-hoc analysis decision. Last, others are proposing statistical approaches to aid in function identification. Hall et al. (2020) proposed the ANSA approach which relies on two nonparametric tests: (1) Mann-Whitney-Wilcoxon to test differences in median between test condition and control and (2) Mann-Kendall test to evaluate trendiness within conditions. Although structured visual analysis advancements have been developed to support visual analysis, Dowdy et al. (2022) identified few studies reported using these structured approaches in experimental studies.

Research on agreement between raters who analyze SCED graphs has shown to be variable. Graph construction’s influence on visual analysts’ decision making is one variable that has been underinvestigated. If specific graphical elements are identified that influence visual analysis, it may inform the construction of graphs in clinical practice and research. Furthermore, visual analysts may be trained to specifically identify these features to inform their visual analysis process. Several reviews have identified inconsistency in graph construction, specifically within behavior analysis (Kubina et al., 2017) and special education (Kubina et al., 2021; Ledford et al., 2019; Peltier et al., 2022a; Peltier, Muharib et al., 2022b).

Two variables with preliminary evidence to suggest they may influence analysts’ decisions when interpreting time-series graphs are the process used to scale the y-axis (i.e., ordinate) and the ratio of the x-axis length to the y-axis height (i.e., data points per x-axis to y-axis ratio [DPPXYR]). Ordinate truncation involves adjusting the scaling of the y-axis to not represent the full possible scale of values given the measurement approach. For example, if interval recording is used the y-min and y-max would be 0% and 100%, respectively. Scaling the y-axis max value to 80% would result in ordinate truncation and enhance Type I errors, meaning effects will be viewed as larger than a graph displaying an untruncated ordinate. The data points per x- to y-axis ratio is conceptualized as considering the length of the x-axis in comparison to the length of the y-axis while also considering the density of the data points plotted along the x-axis: (x-axis length/y-axis length)/number of data points that could be plotted along the x-axis. Readers can view Fig. 1 for a visual of how manipulation of the DPPXYR changes the graphical display. Error rates for y-axis scaling, varied with some reviews identifying less than 5% error (e.g., Peltier et al., 2022a) although others identified slightly higher error rates (i.e., Kubina et al., 2017). A common theme across reviews was the low adherence to recommended x- to y-axis ratios, with values ranging from 4% to 15% adhering to recommendations (Kubina et al., 2017; Kubina et al., 2021; Ledford et al., 2019; Peltier, Muharib et al., 2022b). Some may argue the ratios recommended lack empirical evidence to be recommended, but the dispersion of ratios used across and within graph type may raise additional questions. Peltier and colleagues used a second metric by which to evaluate the axis ratios, the data points per x- to y-axis ratio (DPPXYR; see Radley et al., 2018), and found 93% (Peltier, Muharib et al., 2022b) and 96% (Peltier et al., 2022a) of graphs had ratios outside the recommended boundaries.

**Fig. 1:**
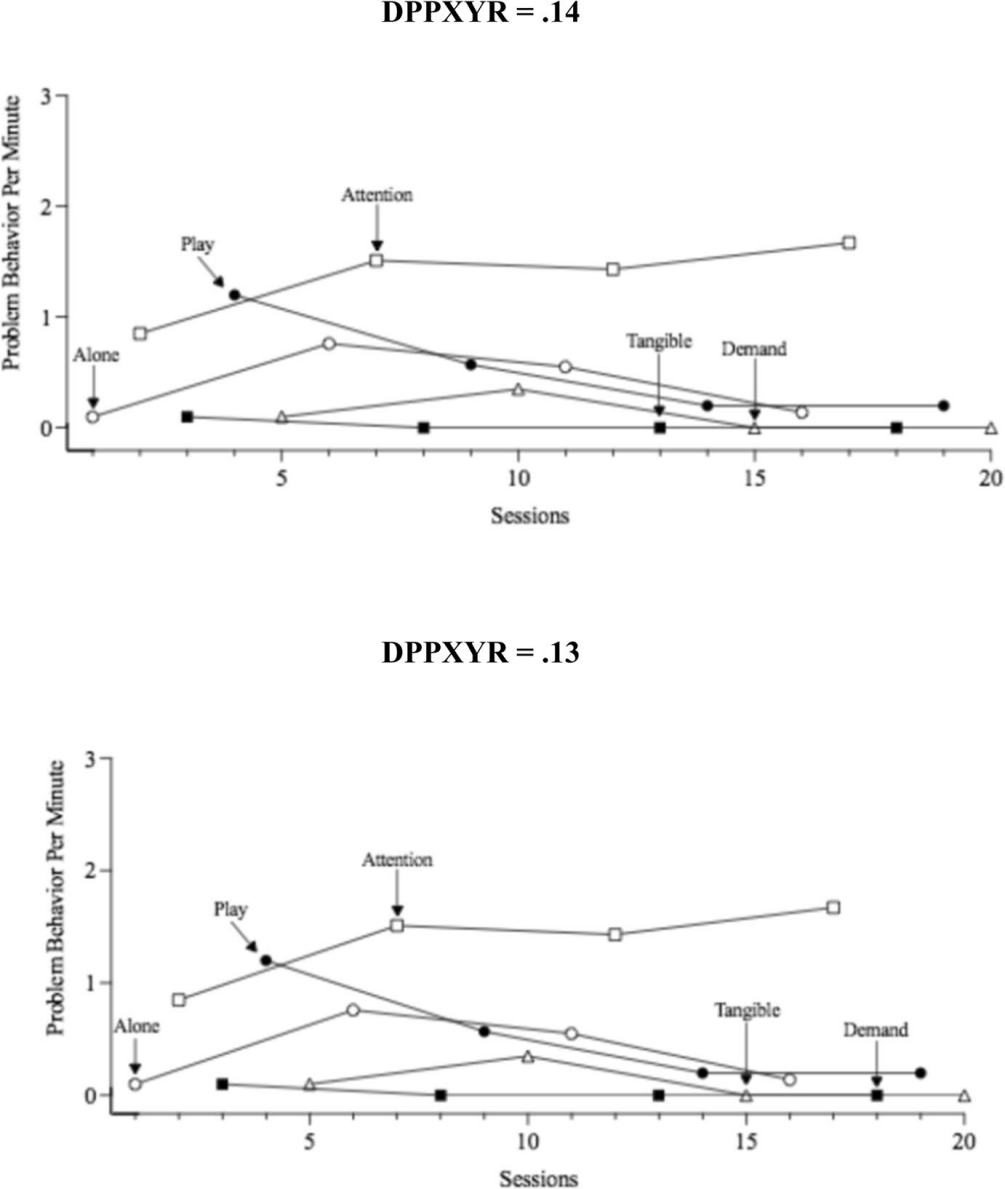

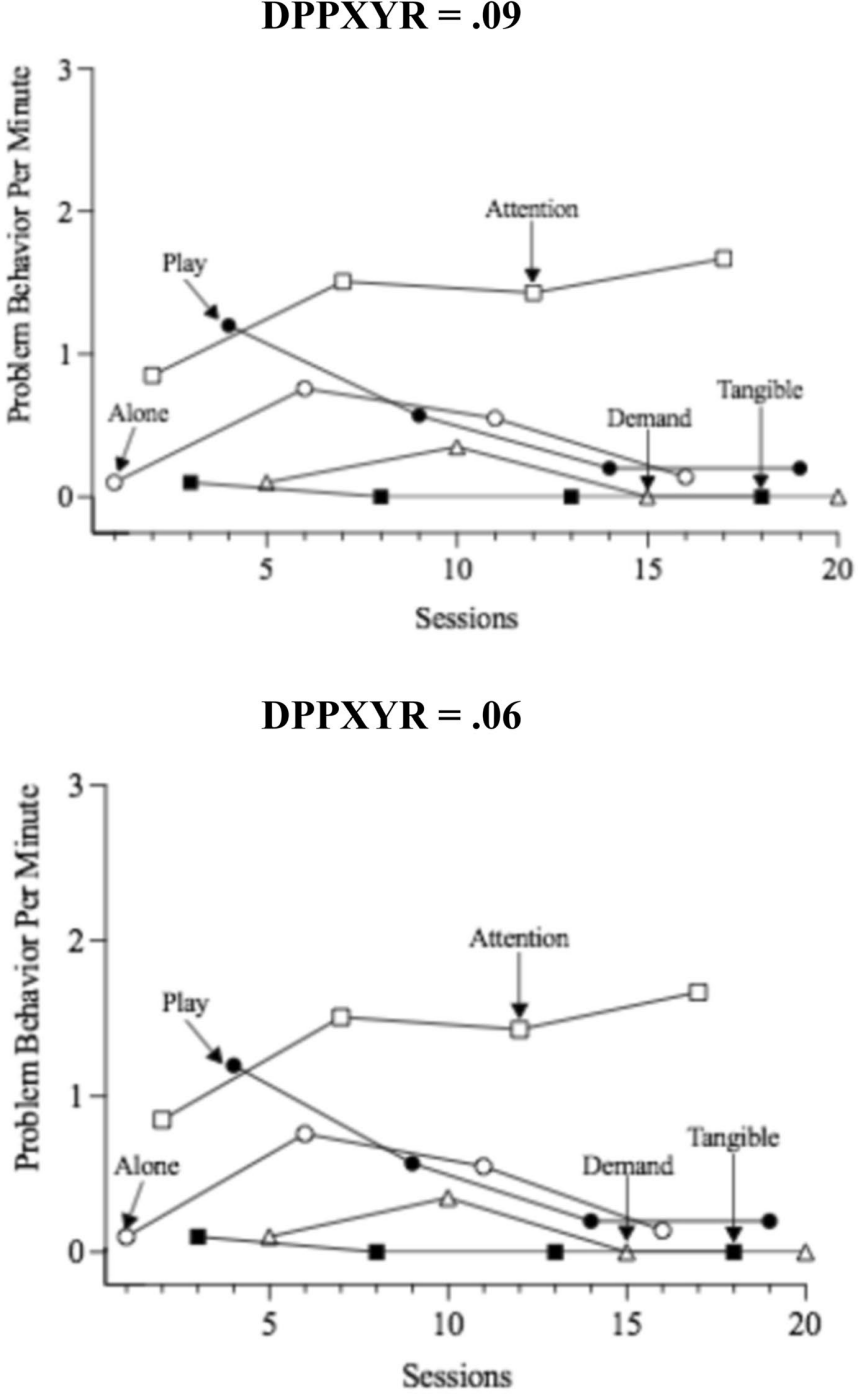
Visualization of DPPXYR manipulation using same data set

These findings may be of concern as an emerging body of literature suggests that simple elements of the graphical display (e.g., y-axis scaling, DPPXYR) can affect judgments made by visual analysts. Dart and Radley (2017) identified visual analysts evaluating ABAB graphs made more Type I errors as the magnitude of the y-axis was truncated (i.e., 80% y-max had 4.7% error and 40% y-max had 21.9%% error). Rate or latency are used frequently in FAs and these measurement approaches do not have a natural upper bound maximum value. In practice, most graphs will be constructed with the y-maximum set at or slightly above the maximum observed value depending on the scale used. Thus, we opted not to investigate the manipulation of the ordinate scale in this article—although it may be a worthwhile investigation for future research. Radley et al. (2018) identified visual analysts evaluating multiple-baseline design graphs had an increased Type I error rate when graphs were constructed with DPPXYR less than 0.14. The DPPXYR is influenced by the ratio of the lengths of the x and y axis along with the density of the data points plotted within the graphical space and this is where variability may be observed. For example, if an analyst has a graph ratio set but continually collects and plots new data to the graph space without adjusting the x to y axis ratio the DPPXYR value will decrease. We opted to investigate this variable given there has not been an investigation of the influence of the DPPXYR on visual analysis of multielement design graphs and the variability that may arise in graph construction for DPPXYR in practice.

Research suggests mixed findings regarding the consistent use of visual analysis to evaluate FA data in behavior assessment and in the assessment of intervention efficacy. Graph construction is one potential variable that may affect visual analysis that has been under investigated. The aim of the project was to investigate how variation in the DPPXYR estimates affect BCBAs evaluation of multielement graphs displaying results from a functional analysis. Specific research questions that guided this study were (1) is there interrater agreement across BCBAs visual analysis to identify a function(s) when DPPXYR is accounted for, and (2) is there alignment between a BCBA’s detected function(s) and modified visual inspection criteria (Roane et al., 2013)?

## Method

### Participants

The target sample for this project were BCBAs with experience in conducting FAs through SCEDs and visual analysis. Other behavior-oriented related personnel, who generally do not make graph-based decisions, were excluded from this research. The certifications excluded from the study were board certified assistant behavior analysts (BCaBAs), state certified behavior specialist, and registered behavior technician (RBT). This sample was selected because the Behavior Analyst Certification Board (BACB) *Task List,* 5^th^ Edition (Behavior Analyst Certification Board, 2017) states BCBAs should have experience with SCED and use the methodology to make data-based decisions (i.e., C-11: Interpret graphed data; D-5: Use single-subject experimental design; BACB, 2017; F-9: Interpret functional assessment data).

To recruit participants, a survey was distributed through a listserv hosted by the BACB. An email was sent to solicit BCBAs and BCBA-Ds, which requested their participation in a research study. In particular, participants were informed that the intent was to observe the decisions that BCBAs make when evaluating FA data through multielement design graphs. The email included a link to a Qualtrics survey and stated the survey would take approximately 20 to 30 min to complete.

The survey was sent to 22,353 BCBA/BCBA-D candidates in the United States with a total of 9,348 candidates opening the email. Of potential participants who opened the email, 571 clicked the URL to access the survey and 126 individuals began the survey. Of these 126 participants, 40 participants were removed because they did not complete all visual analysis items and 27 were removed because they did not complete the demographic items. For this study, participants with missing data were excluded for two primary reasons. First, statistical analysis generally rely on complete data for all variables of interest and missing data generally complicates interpretation and generalizability of results. Second, we suspected that the data were likely not missing completely at random (MCAR), thus including participants with missing data increased the likelihood that bias could be introduced into the analysis. Thus, we retained data from 59 participants who completed all survey items.

Most participants had their BCBA (*N* = 46, 78%) with fewer holding a BCBA-D (*N* = 13, 22%). The mean age of participants was 39 years old (range = 24–69). A majority of participants identified as women (*N* = 44, 75%) with one participant who preferred not to say. A majority of participants identified as white (*N* = 49, 83%), 3 (5%) identified as Black, two identified as (3.4%) Latino/a, two (3.4%) identified as Asian, one (1.7%) identified as Native American, and two (3.4%) preferred not to say. Participants were represented from multiple geographical regions in the United States: Northeast (*N* = 22, 37.2%), West Coast (*N* = 13, 22%), Midwest (*N* = 9, 15%), Southeast (*N* = 8, 13.5%), and Southwest (*N* = 7, 11.8%).

### Visual Analysis Task

Once demographic data were collected, participants visually analyzed 32 multielement graphs that displayed results from a functional analysis. A primary purpose of this study was to examine how the alteration of the DPPXYR (Radley et al., 2018) affects BCBAs’ interpretation of detecting a function of behavior. This metric was selected because the x to y-axis ratio can vary based on the software used to generate the graph. Furthermore, the DPPXYR is influenced by the number of data points plotted on the graph, thus a computer generating x:y ratios within recommendations (e.g., between 8:5 and 3:2) will have different DPPXYR estimates depending on the number of data points plotted on the graph. The DPPXYR was calculated for each graph by extracting the dimensions of both the x and y axis, dividing the x value by the y value, and then dividing the outcome by the number of data points. Participants were shown four different graphs for each of the eight data sets (DPPXYR = 0.06, 0.09, 0.13, and 0.14). These DPPXYR values of 0.06, 0.09, 0.13, and 0.14 were selected by evaluating the data reported in Peltier, Muharib et al. (2022b). Peltier, Muharib et al. (2022b) reviewed a total of 425 graphs that presented data from a multielement design and found the mean DPPXYR was 0.13 (*SD* = 0.18).

The first quartile, median, and third quartile DPPXYR values of the multielement design graphs were used to set the DPPXYR values. We also used 0.14 because this was the minimum recommendation provided by Dart and Radley (2017) to reduce Type I errors. When constructing the four graphs per data set only the DPPXYR was altered—all other graphical elements were held constant and the data remained the same. All graphs were constructed in GraphPad Prism version 9.3.1. This procedure resulted in four variations of each of the eight data sets, yielding a total of 32 graphs. One graph was presented on each page of the survey, and the conditions shown included play, attention, demand, tangible, or alone.

### Graph Construction

Eight data sets were used to create the 32 graphs. Each data set was constructed to adhere to the What Works Clearinghouse (WWC, 2022) criteria version 5.0 for multielement/alternating treatments designs were consulted with to eliminate the potential confound of limited data per conditions. The WWC is a widely recognized resource with rigorous standards for designing and conducting educational research. As such, each data set included 4 data points per condition. Each data set included 20 sessions, in which play, tangible, demand, attention, and alone conditions were presented in a quasi-random order across sessions. Four data points were generated in sets of five representing each of the five conditions using a random number generator function in the R ecosystem called runif(). The parameters included in the random number generator code were as follows: (1) the data point was a continuous outcome, and (2) the data points were bounded based on the y-axis scale. For example, in data set #2 the y-axis represents property destruction per minute and is scaled from 0 to 3. Bounds for scaling were included when generating the data points. Eight distinct graphs were created using this method and no additional graphs were created. Conditions were applied to each data set after the data were graphed and the modified visual inspection (MVI; Roane et al., 2013) was used as an objective approach to identify the function of each multielement design data set. Posted on the corresponding OSF page and named *Multielement Design Graph Sets with Results* is each graph set with the identified function based on MVI. Also posted on the corresponding OSF page is an R Markdown document that includes example code that we used for generating data points.

### Modified Visual Inspection

The secondary purpose of the study was to evaluate the extent to which visual analysts’ responses aligned with MVI. MVI is well-established in the literature for interpreting functional analysis outcomes (e.g., Roane et al., 2013; Wacker et al., 2013; Hagopian et al., 2013), and MVI criteria has been exclusively applied to standard functional analyses that are presented in a multielement design format. For both reasons we selected to use MVI to detect a function of dependent variables. An alignment agreement occurred when the visual analyst identified all of the functions detected using MVI. Partial matches were coded as a disagreement. MVI criterion requires placing an upper and lower criterion line 1 *SD* above and below the mean of all points in the play condition to detect a function. If all of the data points in the play condition are zero, then both criterion lines are placed on this value. The MVI is scored as multiple functions, if more than one condition meets the criteria for differentiation. We refer the reader to the Appendix in Roane et al. (2013) for detailed instructions that also include rules for trends, low-rate behavior, and low magnitude of effects. In summary, MVI enables the ability to assess data point percentages, trends, rates of behavior, and multiple maintaining variables, all in perspective of the criteria lines.

### Interrater Reliability

Interrater agreement (IRA) of MVI was collected on 50% of the graphs developed using the Roane et al. (2013) criteria. The IRA observer held her BCBA and had 11 years of experience developing and analyzing SCED graphs which included functional analyses displayed on multielement design graphs. To calculate MVI, the BCBA read the criteria and the researcher modeled computing the MVI on a separate graph and then provided a different multielement design graph for the observer to compute MVI independently. When an IRA of 100% was obtained during training, the second observer then analyzed a randomly selected sample of 50% of the multielement design graphs included in the survey.

The researcher calculated IRA for the agreement of functions based on the MVI criteria. An agreement was scored if both observers recorded the same function, functions, or lack of function for each graph. Next, the graphs that resulted in agreements were divided by the total number of graphs and multiplied by 100 ([# of interval of 100% agreement]/[total # of intervals] × 100). The IRA for the MVI criteria was 100%.

Next, two additional research team members reviewed all eight data sets to verify the functions based on the MVI criteria. Both researchers held a doctoral degree in either behavior analysis or special education, had a minimum of 10 years of experience in interpreting SCED graphs, and had a minimum of 15 peer-reviewed publications that involved the use of SCED. The IRA for the MVI criteria was 100%.

### Procedure

Before data collection, all study procedures were approved by the affiliated university's Institutional Review Board #28829. Hyperlinks were distributed through the BACB listserv. Individuals who followed the link to participate were initially presented with a consent document explaining the purpose of the study. Potential participants were informed that the study was intended to better understand how BCBAs detect the function of problem behavior. However, due to the purpose of the study, the consent document did not mention manipulation of the DPPXYR. Once participants agreed to consent to the study, they proceeded to the demographic questionnaire. The demographic questionnaire asked questions pertaining to the participants’ experience as a behavior analyst, frequency in use of SCED and interpretation, frequency of conducting and interpreting FAs using multielement designs, and education level. After completing the demographic section, participants were given instructions for the visual analysis task.

For each of the 32 graphs, participants were instructed to complete the following task: A. Evaluate the time-series graphs, B. Identify the function of the behavior and by function we mean the reason or purpose the behavior occurs. C. You may identify one function or more than one function or determine no function can be determined. Graphs were presented to participants in a random order during the visual analysis task. After answering all questions for 32 graphs, the visual analysis task ended, and participants were thanked for their participation. Mean survey completion time was 108 min (*SD* = 539 min) but after excluding an outlier (4,175 min) mean survey completion time was 39 min (*SD* = 396 min). It was possible for participants to start and stop the survey at their convenience and some participants may have opened the tab and not have completed it in an initial sitting.

### Data Analytic Plan

Generalized linear mixed models (GLMM) were built, and models were compared using generalized linear mixed effects regression (Bates et al., 2015). Mixed-effects models are called “mixed” because they are capable of modeling both fixed (slopes and intercepts that do not vary) and random effects (DeHart & Kaplan, 2019). Whereas, a fixed effects model the average trend, random effects model the extent to which these trends vary across designated grouping factors, in this case each analyst. Generalized linear mixed effect regression that relied on maximum likelihood was used to compare models due to the binomial structure of the response variable. The model build included a binomial error distribution and a specifying logit link function that transforms probabilities bounded by 0 and 1 into a continuous unbounded scale called a log odds. Statistical notation for the GLMM is:

$$logit=\left[P\left(y_{jik}=1\left|s_j,w_i\right.\right)\right]=\beta_0+\beta_1x_{kj}+s_j+w_i,$$

In the notation above, j serves as an index for the rater (j = 1, . . ., J*)*; i is an index for an item (i = 1, . . ., I); k is an index for a level of the experimental condition (k = 1 or 2); y_jik_ is the response from rater j and item i at the k^th^ level of a factor; x_kj_ is the independent variable coded as 0 (when k = 1) or 1 (when k = 2), respectively, for person j; β_0_ and β_1_ are the fixed effects parameters for the intercept and the slope, respectively; _sj_ is the rater random effect; and w_i_ is the item random effect. Models were built and compared in the R environment (R Core Team, 2024) by the first author who used lme4 (Bates et al., 2022) and flexplot (Fife, 2022) as the primary packages to fit and analyze the GLMMs.

In the first evaluation, four nested GLMMs were built based on the theory that the DPPXYR may affect the interpretation of a functional analysis outcome when FA graphs are presented in a multielement design format. In a nested design, lower-level units are nested within higher-level units. This implies that observations within the same group or cluster are more similar to each other than they are to observations in other groups or clusters. Model diagnostics favored a crossed model rather than a nested model.

Next , a crossed design GLMM that was driven by theory was built. In a crossed design, multiple grouping factors are intermingled or crossed. This means that each level of one grouping factor can be observed with each level of another grouping factor. In particular, because all raters interpreted a function for all of the graphs, the graphs within each data set likely entailed different degrees of residual variability. To better understand this hypothesis, first, a likelihood ratio test (LRT) was used to determine the random effects structure best supported by the data. In the baseline GLM, rating was included as the dependent variable and an interaction effect between DPPXYR and the Data Set added. Next, a crossed design GLMM was built that included an interaction between the DPPXYR and data set. The crossed design GLMM allowed for a varied mean per rater and allowed for the mean to vary for each intercept per graph. The random effects structure of the baseline model and the crossed design GLMM model were compared using an anova test. The complexity of the random effects structure was evaluated next to understand if the added complexity aided with the model fit. In particular, the crossed GLMM structure was maintained, but the rater and graph slopes were set to vary. The baseline model was then compared to the crossed design GLMM using the LRT test. Last, we plotted individual level variance of predicted accuracy for each DPPXYR estimate across data sets to visually detect differences across data sets. Model comparisons included both visual analysis and comparisons of the Akaike Information Criterion (AIC), the Bayesian Information Criterion (BIC), and other model diagnostics included in Table 1.

**Table 1:** Model comparison of generalized linear model and generalized linear mixed effect models

Model	AIC	BIC	LLR	deviance
*GLM*				
Logistic Regression Model	2527.5	2538.6		
Logistic Regression Model with Interaction	2530.0	2552.2	-1261.0	2522.0
*Nested GLMM*				
Baseline GLMM	2417.7	2428.8	-1206.8	2413.7
Nonrandom Slopes Model	2419.6	2436.2	-1206.8	2413.6
Random Slopes/Random Intercepts Model	2423.5	2451.2	-1206.7	2413.5
Exploratory Model: Interaction Effects	2419.4	2447.1	-1204.7	2409.4
*Crossed GLMM*				
Nonrandom Slopes/Random Intercepts with Interaction	1414.8	1448.0	-701.37	1402.8
Random Slopes/Random Intercepts with Interaction	1408.8	1491.9	-689.39	1378.8
Random Slopes/Random Intercepts without Interaction	1406.1	1478.2	-690.1	1380.1

*GLM* generalized linear model, *GLMM* generalized linear mixed model, *AIC *Akaike information criterion, *BIC* Bayesian information criterion, *LLR* log-likelihood ratio

### Open Science

The replication crisis, the inability for scientific findings to replicate across future studies, has led to intense efforts to enhance the credibility in research efforts (Hantula, 2019). One strategy to promote more credible research is through research practices focused on transparency and openness in the design, execution, and reporting of scientific findings (Hales et al., 2019). These efforts aim to enhance the reproducibility of research, which have been contextualized within the field of behavior science (Laraway et al., 2019). In this research report, we aim to adhere to these practices. First, the research materials used (i.e., the survey) are openly shared on an open-source repository so others can attempt to replicate our findings. Second, the raw data from our study are shared on an open-source repository so others can attempt to replicate our findings—or perhaps use these data to reanalyze them in a different way or use them as part of a broader project. Third, our data analytic code is shared on an open-source repository so (1) readers can confirm results reported align to the analyses conducted; (2) others can attempt to directly replicate our results; or (3) if researchers engage in a replication with a new data set they can replicate our analysis. These items for replication and reproducibility can be accessed on our Open Science Framework page (Link; Dowdy et al., 2024).

## Results

### DPPXYR Impact on Function Detection

Four nested GLMMs and three crossed GLMMs were built and compared to each other and baseline models to investigate IRA across BCBAs detection of identifying a function. Several steps were taken prior to the GLMM build to ensure that mixed modeling was necessary. First, a correlogram of predictor variables were evaluated along with density curves and scatter plots to ensure that predictor variables were not highly correlated. A strong positive or negative correlation (near 1 or -1) shown in the correlogram may suggest multicollinearity exists between predictor variables. That is, as one variable increases the other tends to increase (positive correlation), or as one variable increases the other tends to decrease (negative correlation). The detection of multicollinearity guide decisions about what variables are to be included in the model. No major violations were observed and further information is available in the OSF repository that includes plots and R code.

A generalized linear model (GLM) logistic regression model was built that did not account for clustering effects. The logistic regression model was built to determine if a slope and intercept model that did not vary was sufficient when compared to models that allowed for examination of the condition of interest while taking variability into account across predictor slopes and intercepts. Findings presented below showed that mixed effects models were preferred when compared to the logistic regression model without an interaction effect (AIC = 2527.48, BIC = 2538.56) and with an interaction effect (AIC = 2530.0, BIC = 2552.2). GLMM was used based on these model selection criteria for the GLM.

### Generalized Linear Mixed Effects Model Results

Table 1 shows the comparison of baseline models to more complex GLMMs. Nested and crossed mixed models were built to include both slopes and intercepts that did not vary and random effects. These random effects were included in the model build to account for trends that may vary across levels of grouping factors, in this case the individual visual analysts. Model diagnostics favored a crossed model rather than a nested model.

Crossed GLMM were built to understand the role of predictors included in the project. A crossed GLMM with a random slopes, random intercepts, and an interaction effect was modeled (AIC = 1408.8, BIC = 1448.0). An LRT was conducted to assess the significance of the difference in model fit between the crossed GLMM and baseline GLM with an interaction. The test yielded a Likelihood Ratio of 2.2e-16 with 11 degrees of freedom. The associated p-value was 0.0001. At the 0.05 significance level, the results indicated statistical significance.

To evaluate if added model complexity was necessary the crossed GLMM with slopes that did not vary, random intercepts, and an interaction was modeled (AIC = 1414.8, BIC = 1448.0) and compared to the crossed GLMM with random slopes, random intercepts, and an interaction. Again, an LRT was conducted to assess the significance of the difference in model fit between the random slopes random intercepts crossed GLMM and the nonrandom slopes random intercepts crossed GLMM. The test yielded a Likelihood Ratio of 0.004344 with 9 degrees of freedom. The associated *p*-value was 0.001. The results suggest that the random slopes and random intercept crossed GLMM model performed better at the 0.05 significance level.

**Fig. 2:**
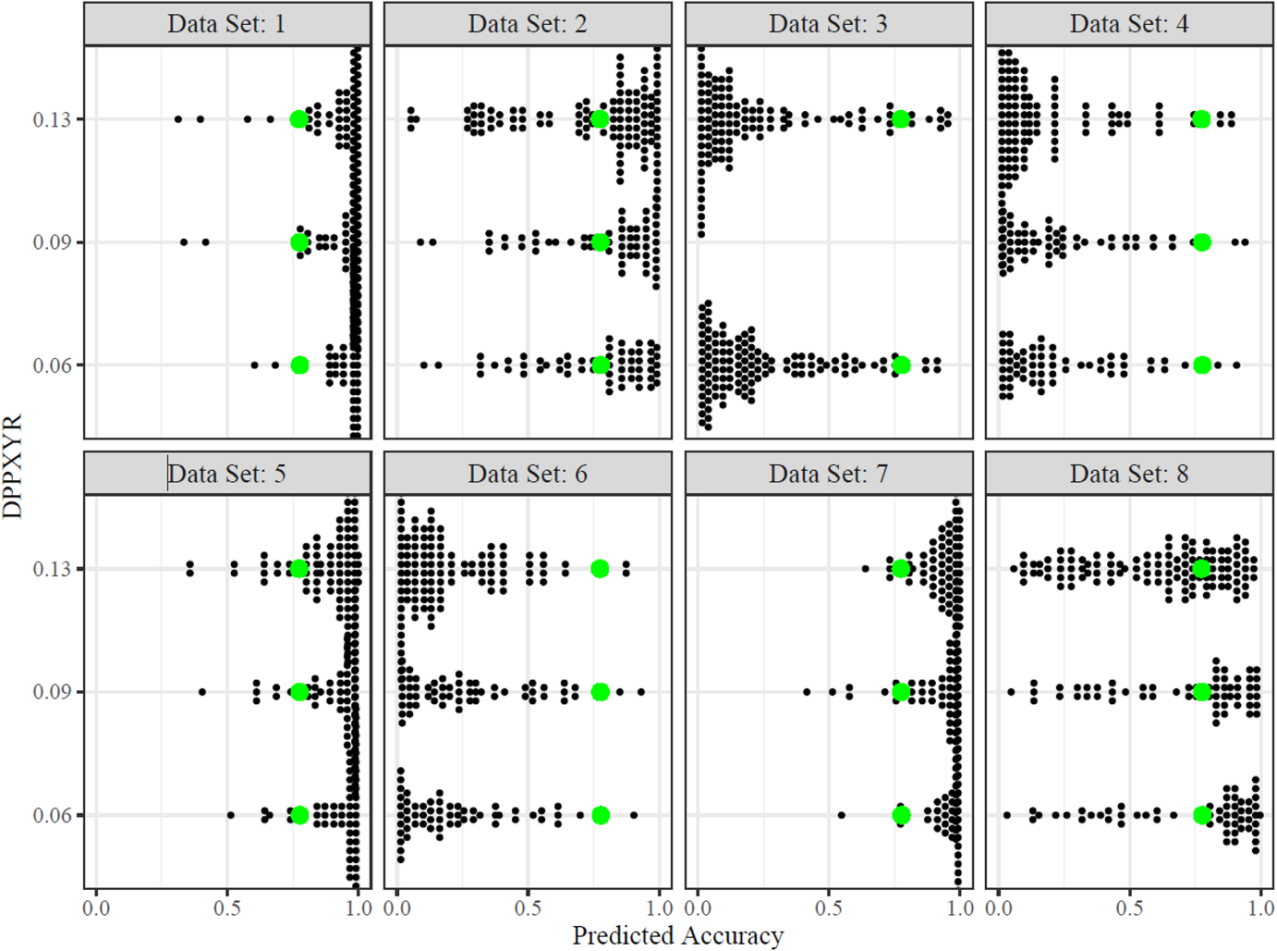
Distribution and Prediction Accuracy of Raters across DPPXYR and Data Sets. Predicted accuracy is based on the crossed GLMM with a random slopes and random intercepts for rater and graph grouping variables

The final crossed GLMM that was built was a random slopes, random intercepts, without an interaction (AIC = 1406.1, BIC = 1478.2) and the model was compared to the crossed GLMM with random slopes, random intercepts, and interaction. An LRT was conducted to assess the significance of the difference in model fit between the random slopes, random intercepts crossed GLMM with and without an interaction. The test yielded a Likelihood Ratio of 0.5111 with 15 degrees of freedom and models were not different at the 0.05 significance level. However, based on AIC and BIC model comparison criteria, the crossed GLMM with random slopes, random intercepts, and without an interaction performed optimally. Figure 2 shows a dot plot of the optimal crossed GLMM model. The green dot shows predicted accuracy per data set across varied DPPXYR metrics for each of the visual analysts’ detection of a function.

### Alignment of Function Detection with Modified Visual Inspection

**Fig. 3:**
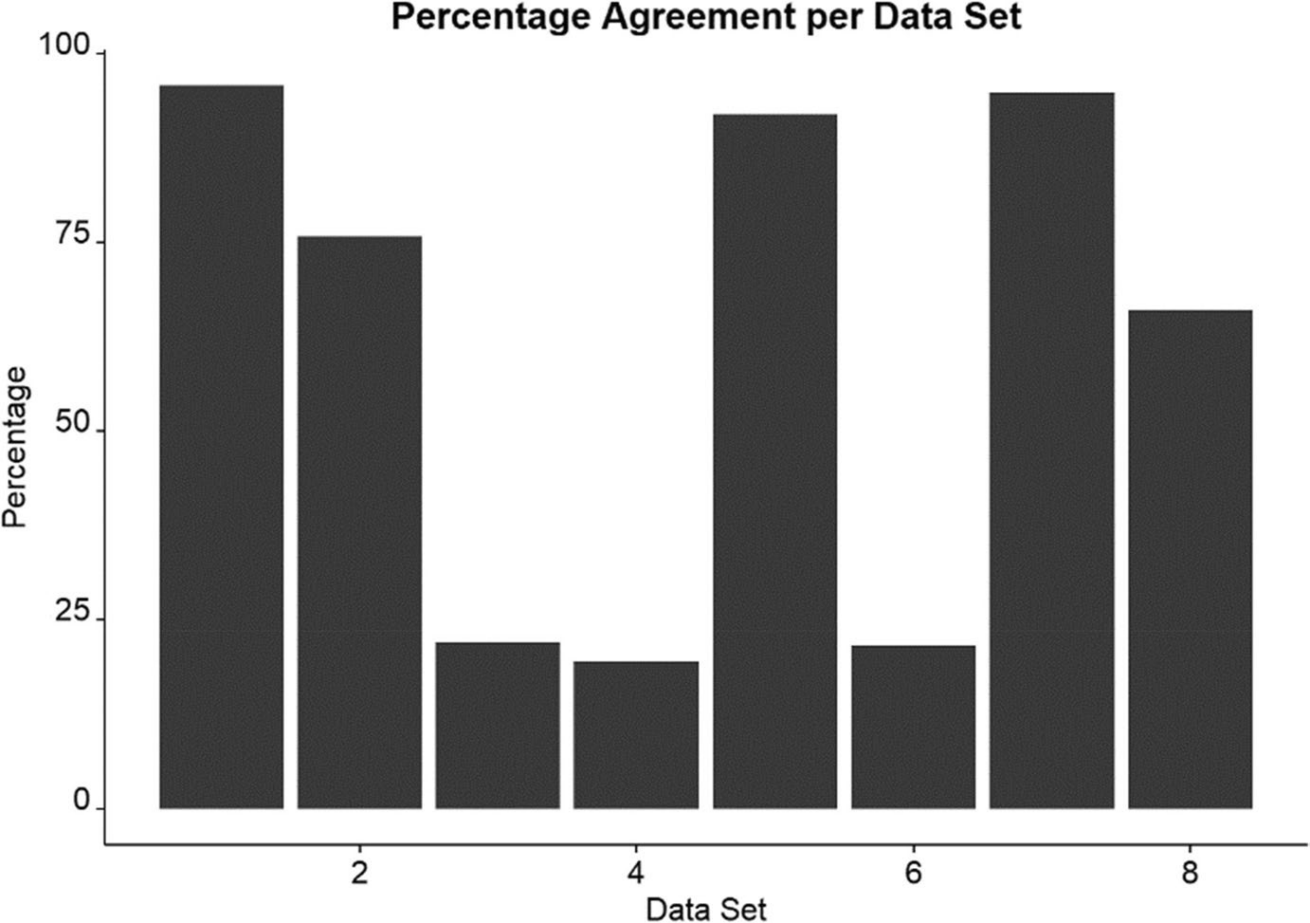
Percentage Agreement between BCBA’s Detection of a Function(s) and Modified Visual Inspection

Next, to evaluate alignment between BCBA’s detection of a function(s) compared to a structured approach to visual analysis, MVI (Roane et al., 2013), percentage agreement across data sets were graphed. Figure 3 shows a bar graph of agreement between BCBA’s detection and MVI. Data sets included in Fig. 3 are identical the multielement design data patterns with varied DPPXYR metrics. Results show that data sets three, four, and six all presented below 25% alignment warranting further investigation. Similar data patterns of graph sets with low alignment included multiple elevated functions with an elevated play condition. Remaining data sets presented above 60% alignment and data sets one, two, five, and six were all around or above 75% alignment. All data sets can be viewed on the Open Science Framework page affiliated with this study.

## Discussion

Our purpose in this study was twofold, first we intended to evaluate how DPPXYR affected BCBAs detection of a function or functions when analyzing functional analysis data presented via a multielement design graph. Our secondary purpose was to evaluate function(s) detection alignment between BCBAs visual analysis and MVI. Results of this study may help us better understand if the DPPXYR may affect behavioral assessment decisions of FAs presented via multielement design graphs, which would ultimately affect function-based intervention selection. In addition, these findings may offer insight on the alignment of BCBAs detection of a function(s) when compared to the MVI. Upon analyzing our findings, we found that DPPXYR metrics of 0.06, 0.09, 0.13, or 0.14 appeared to affect the alignment of raters’ interpretation of a FAs with decisions made through MVI when the slopes and intercepts around the grouping variables rater and graph were set to vary. We also found a rather large distribution between BCBAs detection and lack of their detection of a function(s) when function detection was informed by the MVI. Namely, three of the eight data sets resulted in substantially low correspondence when compared to the other five data sets, highlighting the continuum of complexity around data patch structure in multielement design graphs.

DPPXYR has early evidence that it may affect visual analysis decisions and thus could increase the likelihood of a Type I or Type II error—but specifically for multiple-baseline designs across participants (see Radley et al., 2018). Graph construction can be classified within a family of elements that potentially result in visual analysis detection errors. Other elements that have shown to affect visual analysis include analysts’ varied levels of experience and training (Fisch, 1998; Lanovaz & Hranchuk, 2021), autocorrelation that may affect data interpretation (Jebb et al., 2015), and differences in the data paths being interpreted (Matyas & Greenwood, 1990). Wolfe and Seaman (2023) conducted a secondary data analysis of graphs used in Lanovaz and Hranchuk (2021) to better understand the influence of data characteristics on interrater agreement. Results of Wolfe and Seaman (2023) showed that graphs with a very small or large effect size resulted in higher interrater agreement compared to graphs with moderate level changes and the presence of a trend in the data path adversely affects interrater agreement. Differentiated graph construction elements (i.e., differences in DPPXYR) would not affect the effect size obtained from the raw data, yet it could lead to perceived impact of the intervention based on systematic differences. Our findings appear to align with Wolfe and Seaman (2023) in that graphs with moderate effect sizes, or in the event of a multielement design graph elevated high overlap appeared to have greater disagreement between a structured visual analytic approach and summative visual analysis (Ledford et al., 2018).

Although this was a secondary research question, the alignment between practicing BCBAs and the MVI is a critical finding. One important finding was a common characteristic across data sets when there was high misalignment between BCBAs detection of a function and MVI occurred when there was high overlap between multiple elevated socially maintained data paths and a play condition. These findings align with Danov and Symons (2008) who investigated alignment between visual inspection and a structured approach (Hagopian et al., 1997) and found that rater performance alignment was moderate to low. This is concerning because clinical BCBAs who implement functional analyses likely make intervention decisions that are driven by FA outcomes. Three potential solutions to a varying degree may help reduce the likelihood of Type I and Type II errors when interpreting FA results. These solutions include increased training on visual analysis by applying a structured visual analytic approach and supplementing visual analysis with an effect size to reduce uncertainty.

The findings of the crossed GLMMs support the need to incorporate on-going explicit instruction of visual analysis and structured approaches of visual analysis to BCBAs. Our findings support the need to allocate training efforts around unclear data-paths that are commonly found during visual analysis for BCBAs who may consistently detect the incorrect function of problem behavior. This was shown in the crossed GLMM when random effects were included for the grouping variable rater. In other words, data patterns that result in moderate effect sizes (e.g., Wolfe & Seaman, 2023) compared to large or small effect sizes, and data patterns with high steady responding, overlap, and elevated control or play conditions found in FA multielement designs likely pose greater difficulty in detecting a function.

Several efforts have been made to strengthen visual analysis skills. Retzlaff et al. (2020) created an e-learning module to train registered behavior technicians (RBTs) to implement a structured visual analysis technique named OVI. They found that five of the six participants in the studied mastered applying OVI to data sets during the base training. One participant required a greater dosage of intervention that consisted of the e-learning modules plus feedback, reinforcement of high levels of correct responding, and changing the time of day. Kipfmiller et al. (2019) built a clinical decision-making model using a behavioral systems approach for professional decision-making (Brodhead et al., 2018). The model included four decisions embedded in visual analysis (1) continue intervention; (2) discontinue intervention; (3) modify intervention; or (4) intervention is complete (Kipfmiller et al., 2019). Their results showed that six of eight of the participants increased the percentage of correct responses with the clinical decision-making model alone and two of the eight participants required additional feedback.

Both Retzlaff et al. (2020) and Kipfmiller et al. (2019) focused training efforts on direct care personnel and RBTs; however, our findings suggest that additional training may be necessary to strengthen BCBAs’ visual analysis skill set, particularly when interpreting FA data that are presented in a multielement design format. One potential strategy may be to design training for BCBAs that include graphs with moderate effect sizes (Wolfe & Seaman, 2023). In addition, it may be useful to provide explicit instruction on structured visual analysis (SVA) approaches to assist with clinical decision making, despite the few examples of application found in published applied behavior analytic published research (Dowdy et al., 2022). Explicit training on the selection and application of SVA techniques (e.g., Hagopian et al., 1997; Saini et al., 2018; Wolfe et al., 2016) that includes data sets with moderate effect sizes may strengthen BCBAs clinical judgment. It is notable that as the landscape of functional analysis research continues to evolve, novel methods may lessen difficulty when interpreting FA data and, in turn, DPPXYR may have even less impact on visual inspection. For example, applying the single test function proposed by Hanley et al. (2014) would likely alleviate challenges associated with visual inspection.

Last, it may be useful to supplement visual analysis with an appropriate effect size during the interpretation of SCED graphs. Primary emphasis could be placed on visual analysis during clinical decision making with the effect size used as an additional tool to guide decision making. Dowdy et al. (2021) provide an overview of meta-analyses and effect sizes in applied behavior analysis with primary emphasis on meta-analyzing SCED research to better understand the boundaries of evidence based practices; however, we suggest that effect sizes can be used as an additional tool to supplement VA or SVA during the decision-making process. When applied specifically to multielement designs, several approaches have been recommended to support visual analysis. The ANSA method is a web-based application which includes two nonparametric statistical tests (Hall et al., 2020; Kranak et al., 2021) that aim to compare median performance of target conditions against control (or play) along with evaluating trendiness of data within condition to determine potential function. Other options recommended include the ADISO and ALIV which can be used calculated via a web-based application (http://manolov.shinyapps.io/ATDesign; Manolov & Onghena, 2018). Future research should investigate if VA or SVA with and without estimated effect sizes reduces Type I and Type II errors when interpreting SCED results.

## Limitations and Future Directions

Our results should be considered within the context of several limitations. The aim of our study was to determine how graph construction elements (i.e., varying DPPXYR) may affect the alignment between a BCBA detecting a function(s) in an FA presented in a multielement design format compared to the MVI. Although we found that this graph construction element did not appear to affect interpretation, we do not know the extent to which graph construction may have influenced BCBAs evaluation of the magnitude of separation per condition—thus likely influencing their decision. To address this limitation, future research could incorporate Monte Carlo Simulation techniques to model distributions of visual analysis outcomes using varied graph construction elements (Friedel et al., 2022; Lanovaz & Bailey, 2024).

A second limitation is the response rate to the survey in that the response rate here may not have the *n* necessary to detect small but potentially relevant effects. Despite this limitation, other studies that include visual analyst raters appeared to have evaluated responses from a similar number of raters or less. For example, Wolfe et al. (2016) included 52 experts when evaluating interrater agreement on visual analysis of individual tiers in multiple baseline designs and Diller et al. (2016) included 19 editorial board members when investigating variability, trend, and mean shifts of multielement design data. GLMMs are versatile and can be applied to a variety of study designs, including those with relatively small sample sizes. However, a high response rate is valuable when covariates are added into the design matrix (e.g., Data Set) and the model includes interactions. In theory, the sample size should simultaneously grow with each added covariate to both detect an effect and reduce the risk of a Type II error. An alternative approach often used with relatively small sample sizes is SDT (Rader et al., 2021) and is used to detect participants’ responses to signals or stimuli. A benefit is that SDT focuses on the quality of the decision-making process rather than requiring large sample sizes. Future behavior-analytic graph construction research that includes GLMM could conduct an a priori power analysis and use the analysis to recruit the sample. In addition, future behavior-analytic graph construction research could further explore the use of SDT when analyzing outcomes.

Related to the limited rater response rate, future visual analysis surveys could also include missing survey data into their analysis by using imputation methods. These methods may consist of estimating or predicting the missing data based on the observed data. Imputation could foster the retainment of participant responses while accounting for uncertainty that is often introduced by missing values. In addition, biases introduced by missing data or incomplete data sets could be addressed by assigning different weights to participants based on the inverse of their probability of being included in the analysis. Last, and related to the limited response rate, we did not evaluate additional covariates such as the range of time to complete the survey, experience using visual analysis, and graduate courses taken in SCED. However, all code and data are posted on the corresponding OSF page to allow for additional theory-driven analyses could be conducted.

A third limitation is the sample of respondents we were able to collect data from may not be representative of the actual population we aim to generalize. Although all raters had a BCBA or BCBA-D certificate the heterogenous population of raters coupled with the limited response rate, it is impossible to know how our respondents may differ from the population. However, it is possible, some BCBAs may have seen the focus on visual analysis and closed the survey due to a lack of confidence—thus we may be overestimating alignment. Conversely, BCBAs with a lot of experience and expertise may be extremely busy and declined the offer to participate—thus we may be underestimating alignment. To address this limitation, researchers may consider using crowdsourcing techniques to capture representative samples across distinct groups of individuals to create an aggregate evaluation of the practice (see “manylabs” conversation; Stroebe, 2019).

A final limitation is participants may have recognized the same data set across the different graphical displays (i.e., DPPXYR = 0.06, 0.09, 0.13, 0.14), which could have influenced their response selection. Despite this limitation, other studies that investigated the influence of graph construction have used the exact same procedure (e.g., Dart & Radley, 2017; Kinney et al., 2023; Radley et al., 2018). We also randomized the order that graphs were presented to visual analysts.

## Conclusion

In a recent review of functional analysis of problem behavior Melanson and Fahmie (2023) found that 67.2% of published FAs were presented in a multielement design format. Although this percentage is lower than previous reviews (e.g., Hanley et al., 2003), we suspect that many FAs in practice are graphed using a multielement design. It is notable that when the rater and graph intercepts’ and slopes’ were set to vary as a grouping variable, this resulted in the best model. Suggesting that BCBA’s ability to detect a function is not consistent and graph complexity may affect their ability. Implications from this study may help guide future training efforts of clinical decision making of BCBAs. This is important because a failure to identify a function could prolong the assessment process, and it would be inefficient if such a decision was based on graphical construction or data path complexity in the multielement design graph.

## Data Availability

Supplementary material associated with this article, including the preprint, script code, and data, is available as a project page on the Open Science Framework (Link).
